# Analysis of the association between resolution of disseminated intravascular coagulation (DIC) and treatment outcomes in post-marketing surveillance of thrombomodulin alpha for DIC with infectious disease and with hematological malignancy by organ failure

**DOI:** 10.1186/s12959-020-0216-6

**Published:** 2020-02-07

**Authors:** Noriaki Kawano, Hideo Wada, Toshimasa Uchiyama, Kazuo Kawasugi, Seiji Madoiwa, Naoki Takezako, Kei Suzuki, Yoshinobu Seki, Takayuki Ikezoe, Tsuyoshi Hattori, Kohji Okamoto

**Affiliations:** 1 Department of Internal Medicine, Miyazaki Prefectural Miyazaki Hospital, 5-30 Kitatakamatsu, Miyazaki, 880-8510 Japan; 2 Department of General Medicine, Mie Prefectural General Medical Center, Mie, Japan; 3 Department of Laboratory Medicine, National Hospital Organization Takasaki General Medical Center, Gunma, Japan; 4 Faculty of Medical Technology, Teikyo University, Tokyo, Japan; 5 Department of Clinical Laboratory Medicine, Tokyo Saiseikai Central Hospital, Tokyo, Japan; 6 Department of Hematology, National Hospital Organization Disaster Medical Center, Tokyo, Japan; 7 Emergency and Critical Care Center, Mie University Hospital, Mie, Japan; 8 Department of Hematology, Uonuma Institute of Community Medicine, Niigata University Medical and Dental Hospital, Niigata, Japan; 9 Department of Hematology, Fukushima Medical University, Fukushima, Japan; 10 Department of Medical Affairs, Asahi Kasei Pharma Corporation, Tokyo, Japan; 11 Department of Surgery, Center for Gastroenterology and Liver Disease, Kitakyushu City Yahata Hospital, Fukuoka, Japan

**Keywords:** DIC, Infectious disease, Hematological malignancy, SOFA, Bilirubin, Creatinine

## Abstract

**Background:**

Although disseminated intravascular coagulation (DIC) is life-threatening, any organ failure associated with DIC resolution and outcomes have been unclear.

**Patients and methods:**

A total of 2795 DIC patients (infection: 1990, hematological malignancy: 805) were analyzed in the post-marketing surveillance of thrombomodulin alpha (TM-α). The background factors of sequential organ failure assessment (SOFA) and antithrombin (AT) were investigated in DIC with infectious disease for their association with DIC resolution and outcome using κ statistics, indicating DIC resolution and survival or DIC non-resolution and non-survival. The same analyses were performed for total bilirubin, creatinine, lactate dehydrogenase, and underlying disease in DIC with hematological malignancy.

**Results:**

In DIC with infectious disease, higher SOFA score severity was closely correlated with lower overall survival in both the DIC resolution and non-resolution groups, but AT activity was not. κ coefficients were 0.234, 0.295, and 0.311 for the SOFA score 0–6, 7–12, and 13–24 groups, respectively. In DIC with hematological malignancy, κ coefficients of total bilirubin were 0.251 and 0.434, and those of creatinine were 0.283 and 0.437 in the normal and abnormal groups, respectively, showing better concordance in the abnormal group than in the normal. Other factors had poor concordance.

**Conclusion:**

In DIC with infectious disease, DIC resolution is an important therapeutic target in patients who have higher SOFA score severity. In DIC with hematological malignancy, DIC resolution is similarly important in patients with abnormality of bilirubin and/or creatinine.

**Trial registration:**

The clinical characteristics and treatment outcomes of patients with DIC treated with TM-α between May 2008 and April 2010 were retrospectively analyzed by subgroup analysis of the post-marketing surveillance data.

## Background

Disseminated intravascular coagulation (DIC) is a life-threatening clinical condition with high mortality due to the severe underlying disease, such as sepsis, hematological malignancy, and solid tumors, that is characterized by the systemic activation of coagulation pathways resulting in multiple organ failure [1–5]. Although the mechanism of DIC differs depending on the underlying disease, there is a common process across all cases, characterized by excessive production of thrombi that cause fibrin generation and deposition. Furthermore, fibrinolytic activation and overconsumption of anticoagulation factors can lead to systemic hemorrhage [1–5].

In the pathogenesis of DIC with sepsis, inflammation and coagulation have been closely linked to damage-associated molecular patterns (e.g. high mobility group box 1, histone), pathogen-associated molecular patterns (e.g. lipopolysaccharide), which facilitate secretion of neutrophil extracellular traps from activated neutrophils, and other inflammatory cytokines (e.g. IL-1β, TNF-α) [1–4].

In the pathogenesis of DIC with hematological disease, cancer procoagulant or tissue factor in leukemic cells and tissue plasminogen activator activation have been closely related to activation of excessive production of thrombi and fibrinolytic activation [1–3, 5].

Regarding the diagnosis and treatment of DIC, harmonization of guidelines for DIC was recently performed by the British Committee for Standards in Haematology (BCSH), the Japanese Society of Thrombosis and Hemostasis (JSTH), and the Italian Society for Thrombosis and Haemostasis (SISET) because the recommendations for diagnosis and treatment differed for each of the three guidelines [6]. In Japan, most emergency and hematological physicians make a diagnosis of and provide treatment for DIC according to the diagnostic criteria of the Japanese Association for Acute Medicine (JAAM) for infectious DIC and the Japanese Ministry of Health and Welfare (JMHW) for hematological DIC. Treatment for the underlying diseases of DIC is essential in DIC patients. Furthermore, supportive modalities such as the administration of platelet concentrates, fresh frozen plasma, heparin, and antithrombin play an important role in controlling DIC [6–8].

The hallmark of DIC treatment is the control of inflammation and coagulopathy in DIC with infectious disease and with hematological disease, and thrombomodulin alpha (TM-α) may be an appropriate anticoagulant and anti-inflammatory agent because of its two major effective sites, the lectin-like domain and epidermal growth factor-like domains [3–5]. These sites control inflammation and bind to thrombin to inactivate coagulation activity, forming a complex that activates protein C to generate activated protein C for the control of abnormal coagulopathy [3–5].

A phase 3 study and several retrospective studies including post-marketing surveillance (PMS) have reported the efficacy and safety of TM-α for DIC patients with infectious and hematological diseases [9–12], and it is generally prescribed in clinical practice. Wada H et al. reported the addition of recommendations for the use of TM-α to the “Expert consensus for the treatment of disseminated intravascular coagulation in Japan [13]. However, TM-α is still made no recommendation according to the international guidelines for management of sepsis and septic shock [14]. Furthermore, in the recent RCT, the 28-day mortality rate was not statistical significant but favorable in the TM-α group, including the DIC resolution [15–17].

With respect to DIC resolution, Okuda et al. reported that DIC resolution in patients treated with TM-α was related to a better outcome of DIC [18]. In contrast, Saito et al. reported that higher DIC resolution in patients treated with TM-α was not significantly related to the outcome of DIC [9]. To date, the relationship between DIC resolution and the outcome of DIC has not been fully examined because the etiologies and background of DIC vary in the underlying diseases.

To elucidate the clinical significance of organ failure for the association between DIC resolution and treatment outcome in DIC patients with infectious disease and hematological malignancy, the clinical impact of disease severity of backgrounds on the association between DIC resolution and treatment outcomes was retrospectively analyzed using subgroup analysis of PMS of TM-α. Furthermore, since DIC patients with infectious disease and those with hematological disease have rarely been both studied together, the above issues were analyzed in each subgroup and then discussed with respect to clinical practice.

## Patients and methods

**Fig. 1:**
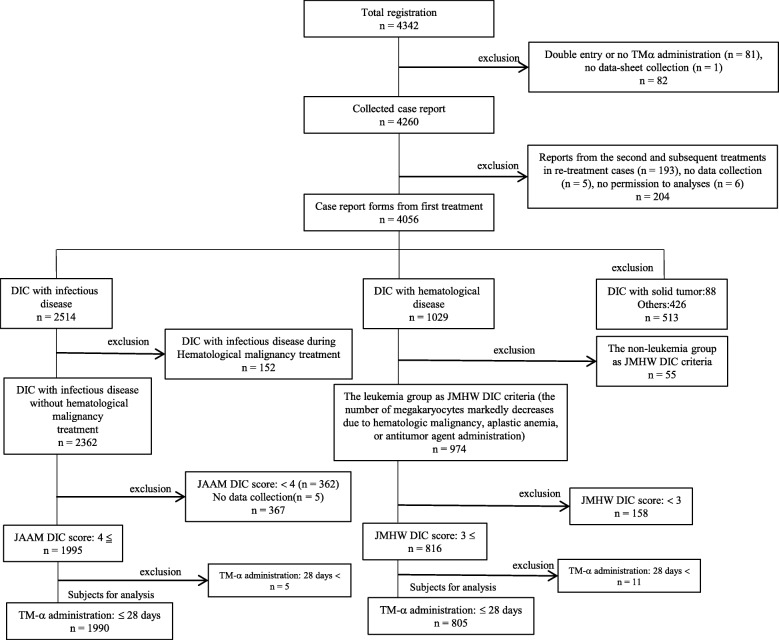
Subject disposition in this PMS study

The clinical characteristics and treatment outcomes of patients with DIC treated with TM-α between May 2008 and April 2010 were retrospectively analyzed by subgroup analysis of the PMS data. Patient selection for the analyses is shown in Fig. 1.

The analyses were conducted with 2795 DIC patients (infection: 1990, hematological malignancy: 805) from the PMS of TM-α who fulfilled the DIC diagnostic criteria of the JAAM for infectious diseases (over 4 points) and the JMHW for hematological diseases (over 3 points).

After treatment with TM-α, resolution of DIC was defined as a score of ≤3 points using the diagnostic criteria of the JAAM and of ≤2 points using those of the JMHW for DIC with infectious and hematological diseases, respectively. Survival at 28 days from the beginning of TM-α treatment or at the end of observation was calculated.

In DIC with infectious disease, the severity of organ failure was assessed by the sequential organ failure assessment (SOFA) score. In hematological malignancy, to assess the severity of organ failure, total bilirubin (T. Bil) and creatinine, which are a subscore of the SOFA because the SOFA score was not clinically applied for DIC with hematological malignancy, were used. Furthermore, lactate dehydrogenase (LDH) was also used for the evaluation of the activity of hematological malignancy.

### Statistical analysis

The following background factors were investigated to examine the association between DIC resolution and treatment outcome using κ statistics [19], indicating the concordance of DIC resolution and survival or DIC non-resolution and non-survival: SOFA and antithrombin (AT) activity for DIC with infectious disease; underlying disease, T. Bil, LDH, and creatinine for DIC with hematological malignancy. The strength of agreement for the κ statistics was described in a previous study [19]: κ < 0.00, poor; 0.00–0.20, slight; 0.21–0.40, fair; 0.41–0.60, moderate; 0.61–0.80, substantial; and 0.81–1.00, almost perfect. It has been noted that these divisions provide useful benchmarks, though they are arbitrary definitions.

In patients with infectious diseases, SOFA severity was divided into 3 groups (SOFA score 0–6, 7–12, and 13–24), and AT activity was divided into 4 groups (AT activity ≤30, 30% < AT activity ≤50, 50% < AT activity ≤70%, and AT activity > 70%). In patients with hematological diseases, the plasma concentrations of T. Bil and creatinine were divided into 2 groups (< 1.2 mg/dL (normal) and ≥ 1.2 mg/dL), the plasma concentration of LDH was divided into 2 groups (≤ 222 U/L (normal) and > 222 U/L), and underlying diseases were divided into 8 groups (acute myeloid leukemia (AML), acute promyelocytic leukemia (APL), acute lymphoid leukemia (ALL), chronic myeloid leukemia, chronic lymphocytic leukemia, myelodysplastic syndromes, multiple myeloma, and malignant lymphoma (ML)). All analyses were performed using SAS ver. 9.4 (SAS Institute, Co. Ltd., Cary, NC, USA) by EPS Corporation (Tokyo, Japan) according to the statistical analysis plan.

This study was conducted in compliance with the guidelines for Good Post-Marketing Surveillance Practices as required by the Japanese Ministry of Health, Labour, and Welfare, and was performed through a contract agreement with the Japanese Society on Thrombosis and Hemostasis and Asahi Kasei Pharma Corporation.

## Results

### Backgrounds of DIC patients

The clinical characteristics of the 1990 DIC patients (848 male patients and 1142 female patients) with infectious diseases are summarized in Table 1. The age of the DIC patients with infectious disease at diagnosis ranged from 0 to 102 years (median age, 71 years). The JAAM DIC scores in the DIC patients with infectious diseases ranged from 4 to 8 points (median score, 6). The SOFA score in the DIC patients with infectious diseases ranged from 1 to 23 points (median score, 10). The AT activity in the DIC patients with infectious diseases ranged from 7.5 to 140% (median score, 55).

**Table 1:** DIC with infectious disease: Patients’ characteristics

Background factor	Median (range) or *n* (%)
Sex, *n* (%)	
Male	848 (42.6)
Female	1142 (57.4)
Age, years	71 (0–102)
DIC duration before TM-α treatment, days	0 (0–51)
Total dose of TM-α, U/kg	369.2 (45–609.5)
Duration of TM-α	6 (1–27)
Prior DIC treatment, n (%)	
+	762 (38.3)
-	1228 (61.7)
Prior heparan sulfate and low molecular weight heparin, *n* (%)	
+	198 (9.9)
-	1792 (90.1)
Prior serine protease inhibitors, *n* (%)	
+	550 (27.6)
-	1440 (72.4)
Prior AT, *n* (%)	
+	375 (18.8)
-	1615 (81.2)
Platelets, 10^4^/μL	5.2 (0–126)
PT ratio	1.32 (0.8–19.06)
FDP, μg/mL	29.2 (0.8–3200)
FBG, mg/dL	358.7 (0.1885–1481)
AT, %	55 (7.5–140)
JAAM DIC score	6 (4–8)
SOFA score	10 (1–23)

The plus and minus signs mean with and without treatment, respectively

*AT* antithrombin, *DIC* disseminated intravascular coagulation, *FBG* fibrinogen, *FDP* fibrin/fibrinogen degradation products, *JAAM* Japanese Association for Acute Medicine, *SOFA* sequential organ failure assessment, *TM-α* recombinant human thrombomodulin

The clinical characteristics of the 805 DIC patients (318 male patients and 487 female patients) with hematological malignancy are summarized in Table 2. The age of the DIC patients with hematological malignancy at diagnosis ranged from 0 to 92 years (median age, 63 years). Their JMHW DIC scores ranged from 3 to 9 points (median score, 4.0).

**Table 2:** DIC with hematological malignancy: Patients’ characteristics

Background factor	Median (range) or *n* (%)
Sex, *n* (%)	
Male	318 (39.5)
Female	487 (60.5)
Age, years	63 (0–92)
DIC duration before TM-α treatment, days	0 (−1–31)
Total dose of TM-α, U/kg	380 (100.6–549)
Duration of TM-α	6 (1–28)
Prior DIC treatment, n (%)	
+	197 (24.5)
-	608 (75.5)
Prior heparan sulfate and low molecular weight heparin, n (%)	
+	101 (12.5)
-	70.4 (87.5)
Prior serine protease inhibitors, *n* (%)	
+	92 (11.4)
-	713 (88.6)
Prior AT, *n* (%)	
+	42 (5.2)
-	763 (94.8)
Platelet, 10^4^/μL	3.25 (0.2–51.9)
PT ratio	1.26 (0.76–7.55)
FDP, μg/mL	48.4 (0.8–1760)
FBG, mg/dL	204 (14–966.5)
AT, %	86.1 (10.2–150)
JMHW DIC score	4 (3–9)

The plus and minus signs mean with and without treatment, respectively

*AT* antithrombin, *DIC* disseminated intravascular coagulation, *FBG* fibrinogen, *FDP* fibrin/fibrinogen degradation products, *JMHW* Japanese Ministry of Health and Welfare, *TM-α* recombinant human thrombomodulin

### κ coefficient, SOFA severity, and overall survival rate with infectious disease

In DIC with infectious disease, the κ coefficient between DIC resolution and treatment outcome was analyzed according to the SOFA severity group (Table 3). The κ coefficients were 0.234, 0.295, and 0.311 in the SOFA score groups 0–6, 7–12, and 13–24, respectively (Table 3). Thus, DIC resolution and DIC non-resolution accorded with survival and non-survival, respectively, in the groups with higher SOFA severity (Table 3).

**Table 3:** Relations between DIC resolution and treatment outcome in DIC with infectious disease

Group	OS, % (n)		κ	
	DIC resolution	DIC non-resolution	Point estimate	95% CI, [lower, upper] limits
SOFA score				
0–6	91.3 (84)	69.6 (48)	0.234 ± 0.067	[0.103, 0.366]
7–12	89.7 (201)	58.5 (161)	0.295 ± 0.035	[0.226, 0.365]
13–24	84.5 (71)	41.8 (107)	0.311 ± 0.043	[0.227, 0.394]
AT activity, %				
≤ 30	77.1 (27)	38.8 (38)	0.301 ± 0.074	[0.156, 0.446]
30 < AT activity ≤50	87.3 (117)	51.2 (134)	0.300 ± 0.039	[0.225, 0.376]
50 < AT activity ≤70	90.9 (169)	53.4 (125)	0.353 ± 0.039	[0.277, 0.429]
> 70	89.6 (112)	54.6 (83)	0.333 ± 0.049	[0.238, 0.428]

The degree of accordance between DIC resolution and treatment outcome according to the SOFA score group was analyzed by κ coefficient in DIC with infectious disease

*CI* confidence interval, *DIC* disseminated intravascular coagulation, *OS* overall survival, *SOFA* sequential organ failure assessment

In particular, the SOFA score 13–24 group with DIC non-resolution showed poor overall survival (OS) (41.8%). Remarkably, the DIC resolution group achieved higher OS at 28 days than the DIC non-resolution group regardless of the SOFA score group in DIC with infectious disease (Table 3).

### κ coefficient, AT activity, and overall survival

Regarding AT activity, the coefficients were not remarkable and were constant among the three groups (Table 3).

The group with AT activity ≤30% with DIC non-resolution showed poor OS (38.8%). Remarkably, the DIC resolution group achieved higher OS at 28 days than the DIC non-resolution group regardless of the AT activity group in DIC with infectious disease (Table 3).

### κ coefficients, underlying disease, and overall survival with hematological malignancy

In DIC with hematological malignancy, the κ coefficient of underlying disease was approximately 0.3 regardless of the group of underlying hematological malignancy (AML, APL, ALL, and ML) (Table 4).

**Table 4:** Relations between DIC resolution and treatment outcome in DIC with hematological malignancy

Group	Underlying disease	OS, % (n)		κ	
		DIC resolution	DIC non-resolution	Point estimate	95% CI, [lower, upper] limits
0	AML	88.1 (104)	62.0 (57)	0.276 ± 0.062	[0.155, 0.397]
1	APL	98.6 (73)	67.9 (36)	0.339 ± 0.071	[0.200, 0.478]
2	ALL	95.3 (61)	70.4 (19)	0.301 ± 0.105	[0.096, 0.506]
3	CML	85.7 (6)	100.0 (4)	−0.170 ± 0.149	[− 0.462, 0.122]
4	CLL	66.7 (4)	50.0 (1)	0.147 ± 0.347	[−0.538, 0.823]
5	MDS	82.4 (14)	36.4 (4)	0.467 ± 0.172	[0.131, 0.804]
6	MM	100.0 (6)	55.6 (5)	0.390 ± 0.177	[0.044, 0.736]
7	ML	90.0 (36)	48.0 (24)	0.400 ± 0.086	[0.232, 0.568]

The degree of accordance between DIC resolution and treatment outcome according to the underlying disease of hematological malignancy was analyzed by κ coefficient in DIC with hematological malignancy

*ALL* acute lymphoid leukemia, *AML* acute myeloid leukemia, *APL* acute promyelocytic leukemia, *CI* confidence interval, *CLL* chronic lymphocytic leukemia, *CML* chronic myeloid leukemia, *DIC* disseminated intravascular coagulation, *MDS* myelodysplastic syndromes, *ML* malignant lymphoma, *MM* multiple myeloma, *OS* overall survival

The DIC resolution group achieved higher OS at 28 days than the DIC non-resolution group regardless of the underlying disease (except for CML) (Table 4).

### κ coefficients, parameters (bilirubin/creatinine/LDH), and overall survival with hematological malignancy

In DIC with hematological malignancy, κ coefficients between DIC resolution and treatment outcome were examined according to T. Bil, creatinine, and LDH levels. The κ coefficients were higher in the abnormal groups of T. Bil and creatinine (Table 5). The κ coefficients for LDH were not remarkable and constant among the groups (Table 5).

**Table 5:** Relations between DIC resolution and treatment outcome in DIC with hematological malignancy

Group	OS, % (n)		κ	
	DIC resolution	DIC non-resolution	Point estimate	95% CI, [lower, upper] limits
Total bilirubin, mg/L				
< 1.2	92.3 (240)	70.3 (104)	0.251 ± 0.046	[0.162, 0.340]
≥ 1.2	90.3 (56)	43.7 (38)	0.434 ± 0.066	[0.306, 0.563]
Creatinine, mg/dL				
< 1.2	92.1 (268)	66.5 (125)	0.283 ± 0.041	[0.203, 0.364]
≥ 1.2	87.2 (41)	41.7 (25)	0.437 ± 0.080	[0.279, 0.594]
LDH, U/L				
≤ 222	96.2 (51)	69.6 (16)	0.322 ± 0.113	[0.102, 0.543]
> 222	91.4 (255)	59.5 (131)	0.336 ± 0.039	[0.260, 0.412]

The degree of accordance between DIC resolution and treatment outcome according to the total bilirubin level was analyzed by κ coefficient in DIC with hematological malignancy

*CI* confidence interval, *DIC* disseminated intravascular coagulation, *LDH* lactate dehydrogenase, *OS* overall survival

The abnormal LDH (> 222 U/L) group with DIC non-resolution showed poor OS (59.5%). The DIC resolution group achieved higher OS at 28 days than the DIC non-resolution group regardless of T. Bil, LDH, and creatinine levels in DIC with hematological malignancy (Table 5).

## Discussion

In previous reports dealing with DIC resolution and treatment outcomes of DIC, different and controversial results were shown because of differences in the patients’ background characteristics [9, 18]. The present study identified three important points by analyzing DIC resolution and survival or DIC non-resolution and non-survival according to the organ failure, as follows. (i) In DIC with infectious disease, a higher concordance of DIC resolution and survival or DIC non-resolution and non-survival was seen in the severe SOFA score group, but this was not seen for AT activity, for which κ values were constant among the groups. (ii) In DIC with hematological malignancy, a higher concordance of DIC resolution and survival or DIC non-resolution and non-survival was seen in the groups with abnormal T. Bil and creatinine levels, but not for LDH. (iii) The DIC resolution group achieved higher OS at 28 days than the DIC non-resolution group, regardless of any group of background factors (except for the underlying disease of CML).

### κ coefficients and SOFA severity and the correlation between SOFA severity and lower overall survival in DIC with infectious disease

Although factors affecting DIC resolution and prognostic factors affecting OS were identified in previous reports [11, 12, 18, 20–26], the clinical significance of the patients’ background characteristics according to the organ disfunction for the association between DIC resolution and treatment outcomes in DIC patients with infectious disease has been unclear. Previously, regarding the SOFA in DIC with infectious disease, Yamakawa et al. reported the benefit of anticoagulant therapy in sepsis for SOFA scores of 13–17 in a nationwide multicenter registry in Japan [27]. Moreover, Nakajima et al. reported an association between SOFA scores and mortality in patients with sepsis during the first week in the Japanese Society of Education for Physicians and Trainees in Intensive Care (JSEPTIC) DIC study. In particular, Nakajima et al. reported the importance of the central nervous system category of SOFA (days 1, 3, 7) and the coagulation category of SOFA (day 7) [28]. The present PMS study clearly showed that, in DIC with infectious disease, the κ coefficient was higher in the severe SOFA score group than in the mild group. Thus, a higher concordance of DIC resolution and survival or DIC non-resolution and non-survival was shown in the severe SOFA score group. Consequently, in DIC with infectious disease, treatment with the target of DIC resolution may be essential to improve OS according to the increased severity of organ dysfunction.

### κ coefficients for T. Bil and creatinine in DIC with hematological malignancy

Previously, regarding prognostic factors for OS in DIC with hematological malignancy [29–31], Bird reported that hyperbilirubinemia (> 18 mg/L) and renal replacement therapy may be prognostic factors affecting OS on univariate analysis in hematological malignancy patients requiring intensive care unit care and intensive care [29]. Moreover, Bird et al. reported mechanical ventilation and ≥ 2 organ failures as prognostic factors for OS on multivariate analysis. The clinical significance of hyperbilirubinemia and abnormal creatinine levels was also reported previously [29]. The cut-offs of hyperbilirubinemia and abnormal creatinine were reported to be > 1.8 mg/dL or > 2.0 mg/dL and > 1.2 mg/dL or > 1.5 mg/dL, respectively [30, 31]. Consistent with these previous reports [29–32], in the present PMS study, the κ coefficients were higher in the groups with abnormal T. Bil and creatinine levels than in those with normal values. Thus, the concordance of DIC resolution and survival or DIC non-resolution and non-survival was greater in the group with abnormal T. Bil and creatinine levels. Consequently, these findings clearly suggest that, in DIC with hematological malignancy, treatment with the target of DIC resolution may be essential to improve OS when there are hyperbilirubinemia and increased creatinine levels.

### Importance of DIC resolution for OS regardless of any background factors in infectious disease and hematological malignancy

MDS, MM and ML showed higher tendency of the degree of accordance between DIC resolution and treatment outcome than those of AML, APL and ALL in Table 5.

As for the liver dysfunction in hematological disease with DIC, Chi S et al. reported that Liver dysfunction in ML such as bilirubin, aminotransferases, serum choline esterase, and albumin levels, were worse in patients with DIC than those without DIC, indicating impaired production of coagulation factors [33]. Furthermore, DIC exerts significantly negative impact on prognosis of non-Hodgkin lymphoma [33]. Similarly, in our present study, the liver dysfunction had significant impact for the degree of accordance between DIC resolution and treatment outcome in ML.

As for the renal dysfunction, Radojevic-Skodric S et al. reviewed that acute renal failure originated from cancer infiltration, drug, dehydration, amyloidosis and others represents a severe complication of different malignancies, that causes significant morbidity and mortality [34]. However, little was known and discussed about the affection of DIC for the renal dysfunction in hematological malignancy [34]. In our present study, the renal dysfunction had significant impact for the degree of accordance between DIC resolution and treatment outcome in hematological malignancy.

Consequently, regarding as the organ dysfunction in hematological malignancy, the hematological malignancy may tend to be complicated with organ dysfunction due to the infiltration of tumors or the infection due to immunodeficient state. Thus, to improve the organ dysfunction, the DIC resolution may be the therapeutic target in DIC with hematological malignancy.

The present analyses clearly demonstrated that DIC resolution had a positive impact on OS at 28 days, especially the patients with organ failure showed well concordance between DIC resolution and OS. Thus, treatment with the target of DIC resolution may be essential to improve OS in DIC with infection and hematological malignancy. Further study is needed to elucidate the real clinical impact of DIC resolution on OS at 28 days.

### Limitation

The laboratory assays were performed in each site or its contracted laboratory, not one central laboratory, because this study is a post marketing surveillance as a clinical practice. The precision management of laboratory data is well performed in Prefectural Association of Medical Technologists.

## Conclusions

In DIC with infectious disease, the κ coefficient in the high SOFA score group may have significance in clinical practice. Similarly, in DIC with hematological malignancy, the strong κ coefficient in the organ failure patients may have an impact in clinical practice. Consequently, the present study clearly demonstrated that the DIC resolution group achieved higher OS at 28 days than the DIC non-resolution group. Finally, DIC resolution can be a possible main target for the treatment of underlying diseases associated with DIC.

## Data Availability

The data that support the findings of this study are available from Asahi Kasei Pharma Corporation but restrictions apply to the availability of these data, which were used under license for the current study, and so are not publicly available.

## Abbreviations

ALL: Acute lymphoid leukemia
AML: Acute myeloid leukemia
APL: Acute promyelocytic leukemia
AT: Antithrombin
CML: Chronic myeloid leukemia
DIC: Disseminated intravascular coagulation
JAAM: Japanese Association for Acute Medicine
JMHW: Japanese Ministry of Health and Welfare
JSEPTIC: Japanese Society of Education for Physicians and Trainees in Intensive Care
LDH: Lactate dehydrogenase
ML: Malignant lymphoma
OS: Overall survival
PMS: Post-marketing surveillance
SOFA: Sequential organ failure assessment
T. Bil: Total bilirubin
TM-α: Thrombomodulin alpha
